# *N*-(4-Methyl­piperazin-4-ium-1-yl)dithio­carbamate sesquihydrate

**DOI:** 10.1107/S1600536812020521

**Published:** 2012-05-12

**Authors:** Anna Mietlarek-Kropidłowska, Jarosław Chojnacki, Paweł Wityk, Miłosz Wieczór, Barbara Becker

**Affiliations:** a Department of Inorganic Chemistry, Chemical Faculty, Gdansk University of Technology, 11/12 G. Narutowicza Str., 80-233 Gdańsk, Poland

## Abstract

In the crystal structure of the title compound, C_6_H_13_N_3_S_2_·1.5H_2_O, weak N—H⋯S inter­actions between the zwitterionic mol­ecules are observed, leading to an extensively folded layered arrangement parallel to (100). There are three crystallographically independent water mol­ecules in the asymmetric unit, which are disordered and only half occupied.

## Related literature

For the synthesis and structures of a series of ^−^S_2_CN*R*-type zwitterionic dithio­carbamic acids, see: Schramm *et al.* (1984 ▶) for *R* = C_3_H_6_NH^+^(Me)_2_; Kokkou *et al.* (1988 ▶) for *R* = C_3_H_6_NH^+^(Et)_2_ and *R*=C_2_H_4_NH^+^(Et)_2_; Stergioudis *et al.* (1989 ▶) for *R*=C_2_H_4_NH^+^(Me)_2_; Yamin *et al.* (2002 ▶) for *R*=C_2_H_4_NH_3_^+^. For structures of dithio­carbamates incorporating a hydrazine-based skeleton, see: Braibanti *et al.* (1969 ▶); Mattes & Füsser (1984 ▶); Kiel *et al.* (1985 ▶). For the synthesis of dithio­carba­mates, see: Coucouvanis (1979 ▶); Hogarth (2005 ▶); Eul *et al.* (1987 ▶); Hulanicki (1967 ▶); Ivanov *et al.* (1999 ▶). For a description of the Cambridge Structural Database, see: Allen (2002 ▶).
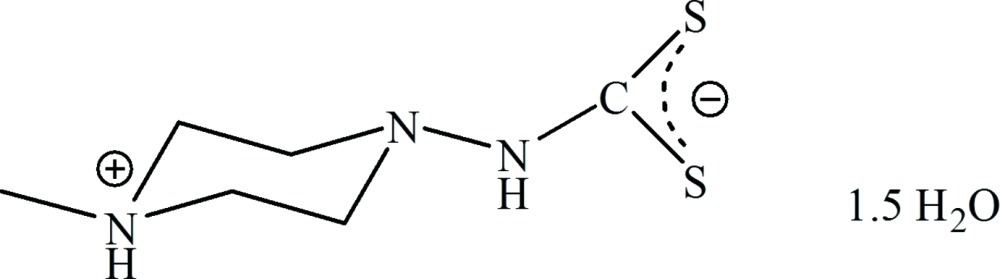


## Experimental

### 

#### Crystal data

C_6_H_13_N_3_S_2_·1.5H_2_O
*M* *_r_* = 218.34
Monoclinic, 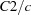

*a* = 23.3560 (18) Å
*b* = 6.8191 (3) Å
*c* = 15.7067 (10) Å
β = 119.920 (9)°
*V* = 2168.2 (2) Å^3^
*Z* = 8
Mo *K*α radiation
μ = 0.46 mm^−1^
*T* = 120 K
0.48 × 0.23 × 0.21 mm

#### Data collection

Kuma KM-4-CCD Sapphire2 diffractometer
Absorption correction: multi-scan (*CrysAlis PRO*; Oxford Diffraction, 2008 ▶) *T*_min_ = 0.952, *T*_max_ = 1
3755 measured reflections
2024 independent reflections
1674 reflections with *I* > 2σ(*I*)
*R*_int_ = 0.021

#### Refinement

*R*[*F* ^2^ > 2σ(*F* ^2^)] = 0.041
*wR*(*F* ^2^) = 0.113
*S* = 1.08
2024 reflections
140 parameters
5 restraints
H atoms treated by a mixture of independent and constrained refinement
Δρ_max_ = 0.60 e Å^−3^
Δρ_min_ = −0.21 e Å^−3^

### 

Data collection: *CrysAlis PRO* (Oxford Diffraction, 2008 ▶); cell refinement: *CrysAlis PRO*; data reduction: *CrysAlis PRO*; program(s) used to solve structure: *SHELXS97* (Sheldrick, 2008 ▶); program(s) used to refine structure: *SHELXL97* (Sheldrick, 2008 ▶); molecular graphics: *ORTEP-3* (Farrugia,1997 ▶) and *Mercury* (Macrae *et al.*, 2006 ▶); software used to prepare material for publication: *WinGX* (Farrugia, 1999 ▶).

## Supplementary Material

Crystal structure: contains datablock(s) global, I. DOI: 10.1107/S1600536812020521/nc2276sup1.cif

Structure factors: contains datablock(s) I. DOI: 10.1107/S1600536812020521/nc2276Isup2.hkl

Supplementary material file. DOI: 10.1107/S1600536812020521/nc2276Isup3.cml

Additional supplementary materials:  crystallographic information; 3D view; checkCIF report

## Figures and Tables

**Table 1:** Hydrogen-bond geometry (Å, °)

*D*—H⋯*A*	*D*—H	H⋯*A*	*D*⋯*A*	*D*—H⋯*A*
N1—H1*N*⋯S2^i^	0.80 (3)	2.60 (3)	3.375 (2)	164 (2)
N3—H3*N*⋯S1^ii^	0.76 (2)	2.67 (2)	3.3131 (18)	143 (2)
N3—H3*N*⋯S2^ii^	0.76 (2)	2.67 (2)	3.2846 (18)	140 (2)

Symmetry codes: (i) 
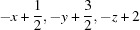
; (ii) 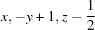.

**Fig. 1.**
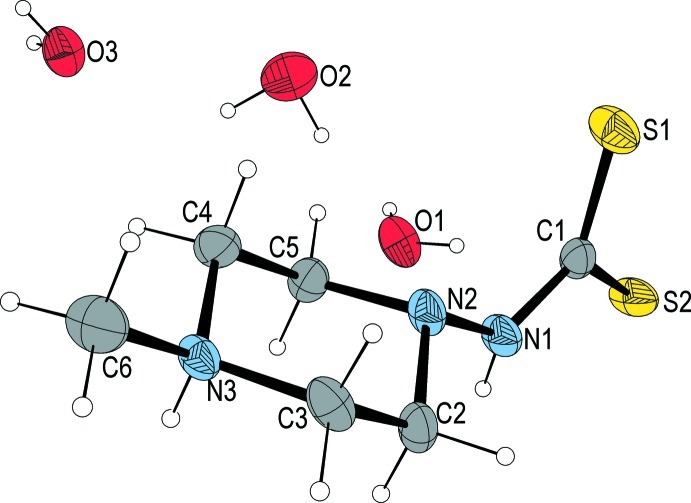
Molecular structure and atom-numbering scheme for the title compound with displacement ellipsoids drawn at 50% probability level. H atoms are represented as arbitrary circles. Only one set of disordered half-occupied water molecules is shown for clarity.

**Fig. 2.**
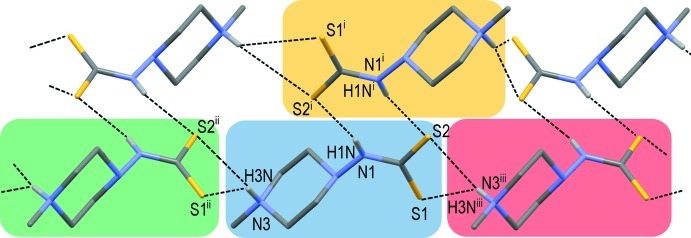
Hydrogen bond network within the crystal of C6N3H13S2×1.5H2O. Dashed lines denote the assumed N—H···S hydrogen bonds. Water molecules are omitted for clarity. [Symmetry codes: (i) 1/2-x, 1.5-y, 2-z; (ii) x, 1-y, -1/2+z); (iii) x, 1-y, 1/2+z.]

## supplementary crystallographic information

### Comment

During our work on the synthesis of organic molecules which may serve as building blocks of more complex structures (*e.g.* coordination compounds) we have focused on dithiocarbamates. These are known to be versatile ligands (Coucouvanis, 1979; Hogarth, 2005) easily bonding to metal soft centres, as well as to be potentially useful chemotherapeutics, pesti- and fungicides (Hulanicki, 1967; Ivanov *et al.*, 1999). Dithiocarbamates (dtc) are amongst the most frequently used bidentate sulfur ligands. More than 2500 compounds with at least one such dtc group can be found in the Cambridge Structural Database (Version 5.33, Nov. 2011, updated to Feb. 2012; Allen, 2002). However, the number of structurally characterized dithiocarbamic acids (invariably present in a form of zwitterionic species) is surprisingly small. These include compounds of ^-^S_2_CNR type with *R* = C_3_H_6_NH^+^(Me)_2_ (Schramm *et al.*, 1984), *R* = C_3_H_6_NH^+^(Et)_2_ and *R*=C_2_H_4_NH^+^(Et)_2_ (Kokkou *et al.*, 1988), *R*=C_2_H_4_NH^+^(Me)_2_ (Stergioudis *et al.*, 1989), *R*=C_2_H_4_NH_3_^+^ (Yamin *et al.*, 2002) and *N*-acetimidoyl dithiocarbamic acid (Eul *et al.*, 1987). Here we describe the structure of new, 1-(4*H*,4-methylpiperazinium)dithiocarbamate sesquihydrate, the first zwitterionic species with N—N bond. The only other structurally characterized dithiocarbamates incorporating hydrazine-based skeleton are salts with potassium (Mattes & Füsser, 1984; Kiel *et al.*, 1985) or hydrazinium (Braibanti *et al.*, 1969) cations.

There are no significant differences in NCS_2_ group geometry compared to other compounds of this type. The notable feature of the title compound is the presence of the intermolecular interactions (Table 1). Eeach molecule of the title compound serves to its close neighbors as a hydrogen bond donor (*via* N—H groups) and acceptor (*via* S atoms, see Figure 2). As a result, all of the NH-groups are engaged in the formation of the network of N—H···S interactions between the pairs of antiparallel chains what leads to the extensively folded layered arrangement observed within the crystal. Additional water molecules are present in vicinity of the twofold axis and are disordered (see experimental refinement section for details).

### Experimental

A stoichiometric amount (0.325 g, 0.26 cm^3^) of carbon disulfide, CS_2_, was added dropwise to a methanol/H_2_O (10:1, *v*/*v*) solution containing 0.5 g (0.52 cm^3^) 1-amino-4-methylpiperazine and 0.24 g potassium hydroxide. The mixture had been stirred for *ca* 25 min until a white precipitate appeared. The clear filtrate was then left at temperature of 5°C for crystallization. After 5 days, well shaped, colorless needle-like crystals suitable for X-ray analysis were collected. Then, the mother liquor was concentrated and after few days more product was isolated. The overall yield was *ca* 50%. The presence of O—H groups was confirmed by FTIR analysis of single crystals taken from the mother liquor using Mattson Genesis II Gold spectrometer equipped with Momentum Microscope as detector (a broad maximum of absorption at 3434 cm^-1^ together with a sharp one at 3239 cm^-1^). However, the product, when taken from the mother liquor and dried using the filter paper, changes - becomes at first opaque and finally takes the form of a powder (most probably because of the removal of the solvent molecules). The microanalysis of such product was also conducted using Vario El Cube CHNS, Elementar (found: %H 6.55, %N 17.04, %C 29.82; calc. for C_6_H_13_N_3_S_2_.1.5H_2_O: %H 7.38, %N 19.24, %C 33.00). The melting point for the title compound (for 10 °/min heating rate) was determined to be 129°C.

### Refinement

All C—H atoms were placed in calculated positions (methyl H atoms allowed to rotate but not to tip) and refined as riding on their carrier atoms with respective bond lengths and *U*_iso_(H) values: C—H = 0.96 Å (CH_3_) and *U*_iso_(H) = 1.5 *U*_eq_(C), C—H = 0.97 Å (CH_2_) and *U*_iso_(H) = 1.2 *U*_eq_(C). Refirement of N—H was carried out withouth restrains.

After refinement of the zwitterionic molecule three electron density peaks are observed that were assigned to three disordered and half-occupied oxygen atoms with distances O1—O2 and O2—O3 2.734 (5) and 2.845 (5) Å, respectively. The symmetry equivalent atoms constitute the second disorder part. The connectivity table was adjusted by using PART -1 *SHELX* instruction to avoid creating bonds to symmetry equivalent oxygen atoms (generated by the twofold axis). Hydrogen atoms bound to O1 and O2 are directed towards acceptor atoms and could have been found and refined as restrained. Positions of H3C and H3D hydrogen atoms were restrained to target 0.85 (2) Å O—H bond lengths and to 1.300 Å H3C—H3D distance to maintain proper H—O—H valence angle.

### Crystal data

**Table:**

C_6_H_13_N_3_S_2_·1.5H_2_O	*F*(000) = 936
*M**_r_* = 218.34	*D*_x_ = 1.338 Mg m^−^^3^
Monoclinic, *C*2/*c*	Melting point: 402 K
Hall symbol: -C 2yc	Mo *K*α radiation, λ = 0.71073 Å
*a* = 23.3560 (18) Å	Cell parameters from 2050 reflections
*b* = 6.8191 (3) Å	θ = 2.6–28.7°
*c* = 15.7067 (10) Å	µ = 0.46 mm^−^^1^
β = 119.920 (9)°	*T* = 120 K
*V* = 2168.2 (2) Å^3^	Block, colourless
*Z* = 8	0.48 × 0.23 × 0.21 mm

### Data collection

**Table:**

Kuma KM-4-CCD Sapphire2 diffractometer	2024 independent reflections
Radiation source: fine-focus sealed tube	1674 reflections with *I* > 2σ(*I*)
Graphite monochromator	*R*_int_ = 0.021
Detector resolution: 8.1883 pixels mm^-1^	θ_max_ = 25.5°, θ_min_ = 2.7°
ω scans	*h* = −18→28
Absorption correction: multi-scan (*CrysAlis PRO*; Oxford Diffraction, 2008)	*k* = −8→7
*T*_min_ = 0.952, *T*_max_ = 1	*l* = −19→11
3755 measured reflections	

### Refinement

**Table:**

Refinement on *F*^2^	Primary atom site location: structure-invariant direct methods
Least-squares matrix: full	Secondary atom site location: difference Fourier map
*R*[*F*^2^ > 2σ(*F*^2^)] = 0.041	Hydrogen site location: inferred from neighbouring sites
*wR*(*F*^2^) = 0.113	H atoms treated by a mixture of independent and constrained refinement
*S* = 1.08	*w* = 1/[σ^2^(*F*_o_^2^) + (0.0755*P*)^2^] where *P* = (*F*_o_^2^ + 2*F*_c_^2^)/3
2024 reflections	(Δ/σ)_max_ = 0.001
140 parameters	Δρ_max_ = 0.60 e Å^−^^3^
5 restraints	Δρ_min_ = −0.21 e Å^−^^3^

### Special details

**Table:**

Experimental. Absorption correction: CrysAlis PRO (Oxford Diffraction, 2008). Empirical absorption correction using spherical harmonics, implemented in SCALE3 ABSPACK scaling algorithm.
Geometry. All s.u.'s (except the s.u. in the dihedral angle between two l.s. planes) are estimated using the full covariance matrix. The cell s.u.'s are taken into account individually in the estimation of s.u.'s in distances, angles and torsion angles; correlations between s.u.'s in cell parameters are only used when they are defined by crystal symmetry. An approximate (isotropic) treatment of cell s.u.'s is used for estimating s.u.'s involving l.s. planes.
Refinement. Refinement of *F*^2^ against ALL reflections. The weighted *R*-factor *wR* and goodness of fit *S* are based on *F*^2^, conventional *R*-factors *R* are based on *F*, with *F* set to zero for negative *F*^2^. The threshold expression of *F*^2^ > 2σ(*F*^2^) is used only for calculating *R*-factors(gt) *etc*. and is not relevant to the choice of reflections for refinement. *R*-factors based on *F*^2^ are statistically about twice as large as those based on *F*, and *R*- factors based on ALL data will be even larger.

### Fractional atomic coordinates and isotropic or equivalent isotropic displacement parameters (Å^2^)

**Table:**

	*x*	*y*	*z*	*U*_iso_*/*U*_eq_	Occ. (<1)
S1	0.11286 (3)	0.40172 (9)	1.02811 (4)	0.0315 (2)	
S2	0.21612 (3)	0.71645 (9)	1.10583 (4)	0.0325 (2)	
N1	0.18466 (9)	0.5160 (3)	0.94834 (12)	0.0253 (4)	
H1N	0.2097 (12)	0.592 (4)	0.9456 (16)	0.030*	
N2	0.14708 (9)	0.3938 (3)	0.86627 (11)	0.0228 (4)	
N3	0.10268 (9)	0.2586 (3)	0.67082 (12)	0.0219 (4)	
H3N	0.1226 (12)	0.321 (4)	0.6552 (17)	0.026*	
C1	0.17032 (10)	0.5395 (3)	1.02061 (13)	0.0237 (5)	
C2	0.19188 (11)	0.2838 (3)	0.84347 (14)	0.0262 (5)	
H2A	0.2237	0.2077	0.9017	0.031*	
H2B	0.2172	0.3759	0.8260	0.031*	
C3	0.15206 (11)	0.1465 (3)	0.75862 (14)	0.0267 (5)	
H3A	0.1820	0.0742	0.7422	0.032*	
H3B	0.1289	0.0497	0.7777	0.032*	
C4	0.06052 (11)	0.3844 (3)	0.69519 (14)	0.0267 (5)	
H4A	0.0324	0.3003	0.7107	0.032*	
H4B	0.0312	0.4664	0.6377	0.032*	
C5	0.10291 (10)	0.5152 (3)	0.78231 (13)	0.0237 (5)	
H5A	0.1291	0.6054	0.7657	0.028*	
H5B	0.0744	0.5950	0.7989	0.028*	
C6	0.06125 (13)	0.1260 (4)	0.58698 (16)	0.0375 (6)	
H6A	0.0899	0.0443	0.5725	0.056*	
H6B	0.0321	0.2046	0.5290	0.056*	
H6C	0.0345	0.0420	0.6042	0.056*	
O1	0.1228 (2)	0.9907 (5)	0.9238 (3)	0.0331 (8)	0.50
H1A	0.1408	0.9093	0.9682	0.050*	0.50
H1B	0.1038	1.0904	0.9270	0.050*	0.50
O2	−0.00204 (19)	0.8579 (6)	0.8020 (3)	0.0426 (8)	0.50
H2C	−0.027 (3)	0.891 (10)	0.742 (2)	0.064*	0.50
H2D	0.0350 (19)	0.920 (9)	0.834 (4)	0.064*	0.50
O3	−0.0827 (2)	0.9861 (6)	0.6047 (3)	0.0323 (8)	0.50
H3C	−0.1054	0.8903	0.5705	0.048*	0.50
H3D	−0.1127	1.0731	0.5844	0.048*	0.50

### Atomic displacement parameters (Å^2^)

**Table:**

	*U*^11^	*U*^22^	*U*^33^	*U*^12^	*U*^13^	*U*^23^
S1	0.0393 (4)	0.0359 (3)	0.0276 (3)	−0.0079 (3)	0.0229 (3)	−0.0077 (2)
S2	0.0284 (3)	0.0499 (4)	0.0237 (3)	−0.0104 (3)	0.0164 (2)	−0.0164 (2)
N1	0.0264 (9)	0.0334 (11)	0.0183 (8)	−0.0050 (9)	0.0127 (7)	−0.0060 (7)
N2	0.0284 (9)	0.0253 (9)	0.0162 (8)	0.0016 (8)	0.0123 (7)	−0.0031 (6)
N3	0.0251 (10)	0.0253 (9)	0.0183 (8)	−0.0077 (8)	0.0130 (7)	−0.0053 (7)
C1	0.0226 (11)	0.0309 (11)	0.0170 (9)	0.0063 (9)	0.0094 (8)	0.0007 (8)
C2	0.0302 (12)	0.0306 (11)	0.0176 (9)	0.0090 (10)	0.0117 (9)	0.0012 (8)
C3	0.0385 (13)	0.0234 (10)	0.0258 (10)	0.0027 (10)	0.0217 (10)	0.0003 (9)
C4	0.0221 (11)	0.0350 (12)	0.0240 (10)	0.0003 (10)	0.0123 (9)	−0.0060 (9)
C5	0.0242 (10)	0.0247 (11)	0.0206 (10)	0.0033 (9)	0.0101 (8)	−0.0027 (8)
C6	0.0364 (13)	0.0467 (15)	0.0334 (12)	−0.0175 (12)	0.0204 (11)	−0.0225 (11)
O1	0.053 (2)	0.0193 (16)	0.0324 (19)	−0.001 (2)	0.025 (2)	−0.0003 (14)
O2	0.035 (2)	0.040 (2)	0.048 (2)	−0.0038 (17)	0.0176 (18)	−0.0047 (17)
O3	0.040 (2)	0.0276 (18)	0.0301 (19)	0.005 (2)	0.0184 (18)	0.0021 (14)

### Geometric parameters (Å, º)

**Table:**

S1—C1	1.690 (2)	C4—C5	1.516 (3)
S2—C1	1.721 (2)	C4—H4A	0.9900
N1—C1	1.344 (2)	C4—H4B	0.9900
N1—N2	1.413 (2)	C5—H5A	0.9900
N1—H1N	0.80 (3)	C5—H5B	0.9900
N2—C5	1.460 (2)	C6—H6A	0.9800
N2—C2	1.470 (3)	C6—H6B	0.9800
N3—C6	1.489 (3)	C6—H6C	0.9800
N3—C4	1.492 (3)	O1—O2	2.724 (5)
N3—C3	1.493 (3)	O1—H1A	0.8236
N3—H3N	0.76 (2)	O1—H1B	0.8269
C2—C3	1.510 (3)	O2—O3	2.845 (5)
C2—H2A	0.9900	O2—H2C	0.86 (2)
C2—H2B	0.9900	O2—H2D	0.86 (2)
C3—H3A	0.9900	O3—H3C	0.8435
C3—H3B	0.9900	O3—H3D	0.8498
			
C1—N1—N2	122.54 (18)	N3—C4—H4A	109.5
C1—N1—H1N	117.8 (17)	C5—C4—H4A	109.5
N2—N1—H1N	118.1 (17)	N3—C4—H4B	109.5
N1—N2—C5	109.09 (16)	C5—C4—H4B	109.5
N1—N2—C2	109.24 (16)	H4A—C4—H4B	108.1
C5—N2—C2	109.73 (14)	N2—C5—C4	109.32 (17)
C6—N3—C4	110.78 (17)	N2—C5—H5A	109.8
C6—N3—C3	111.58 (18)	C4—C5—H5A	109.8
C4—N3—C3	111.21 (15)	N2—C5—H5B	109.8
C6—N3—H3N	106.9 (18)	C4—C5—H5B	109.8
C4—N3—H3N	110.4 (19)	H5A—C5—H5B	108.3
C3—N3—H3N	105.7 (19)	N3—C6—H6A	109.5
N1—C1—S1	122.25 (16)	N3—C6—H6B	109.5
N1—C1—S2	114.87 (16)	H6A—C6—H6B	109.5
S1—C1—S2	122.86 (11)	N3—C6—H6C	109.5
N2—C2—C3	109.37 (18)	H6A—C6—H6C	109.5
N2—C2—H2A	109.8	H6B—C6—H6C	109.5
C3—C2—H2A	109.8	O2—O1—H1A	106.6
N2—C2—H2B	109.8	O2—O1—H1B	84.1
C3—C2—H2B	109.8	H1A—O1—H1B	124.5
H2A—C2—H2B	108.2	O1—O2—O3	123.96 (18)
N3—C3—C2	110.47 (17)	O1—O2—H2C	126 (4)
N3—C3—H3A	109.6	O3—O2—H2D	114 (4)
C2—C3—H3A	109.6	H2C—O2—H2D	116 (6)
N3—C3—H3B	109.6	O2—O3—H3C	108.7
C2—C3—H3B	109.6	O2—O3—H3D	126.7
H3A—C3—H3B	108.1	H3C—O3—H3D	99.4
N3—C4—C5	110.62 (17)		
			
C1—N1—N2—C5	101.4 (2)	C4—N3—C3—C2	−53.9 (2)
C1—N1—N2—C2	−138.6 (2)	N2—C2—C3—N3	58.0 (2)
N2—N1—C1—S1	8.7 (3)	C6—N3—C4—C5	178.46 (17)
N2—N1—C1—S2	−172.63 (15)	C3—N3—C4—C5	53.8 (2)
N1—N2—C2—C3	177.53 (16)	N1—N2—C5—C4	−177.72 (15)
C5—N2—C2—C3	−62.9 (2)	C2—N2—C5—C4	62.6 (2)
C6—N3—C3—C2	−178.18 (17)	N3—C4—C5—N2	−57.9 (2)

### Hydrogen-bond geometry (Å, º)

**Table:**

*D*—H···*A*	*D*—H	H···*A*	*D*···*A*	*D*—H···*A*
N1—H1*N*···S2^i^	0.80 (3)	2.60 (3)	3.375 (2)	164 (2)
N3—H3*N*···S1^ii^	0.76 (2)	2.67 (2)	3.3131 (18)	143 (2)
N3—H3*N*···S2^ii^	0.76 (2)	2.67 (2)	3.2846 (18)	140 (2)

Symmetry codes: (i) −*x*+1/2, −*y*+3/2, −*z*+2; (ii) *x*, −*y*+1, *z*−1/2.
